# Preparation and Characterization of Date Palm Bio-Oil Modified Phenolic Foam

**DOI:** 10.3390/polym16070955

**Published:** 2024-03-31

**Authors:** Paprayil Reghunadh Sarika, Paul Nancarrow, Yassir Makkawi, Taleb H. Ibrahim

**Affiliations:** Department of Chemical and Biological Engineering, American University of Sharjah, Sharjah P.O. Box 26666, United Arab Emirates; sreghunadh@aus.edu (P.R.S.); ymakkawi@aus.edu (Y.M.); italeb@aus.edu (T.H.I.)

**Keywords:** date palm bio-oil, phenolic foam, mechanical properties, thermal conductivity, toughened phenolic foam

## Abstract

In this work, the potential of biomass-derived date palm bio-oil as a partial substitute for phenol in the phenolic resin was evaluated. Date palm bio-oils derived from date palm were used for the partial substitution of phenol in the preparation of phenolic foam (PF) insulation materials. Date palm waste material was processed using pyrolysis at 525 °C to produce bio-oil rich in phenolic compounds. The bio-oil was used to partially replace phenol in the synthesis of phenolic resin, which was subsequently used to prepare foams. The resulting changes in the physical, mechanical, and thermal properties of the foams were studied. The substituted foams exhibited 93%, 181%, and 40% improvement in compressive strength with 10%, 15%, and 20% bio-oil substitution, respectively. Due to the incorporation of biomass waste material, the partial reduction in phenol uses, and the favorable properties, the date palm bio-oil substituted phenolic foams are considered more environmentally benign alternatives to traditional phenolic foams.

## 1. Introduction

Thermal insulation materials are used extensively in buildings and vehicles due to their ability to retain thermal energy and, thus, minimize energy consumption. In general, all thermal insulation materials used in construction must have low thermal conductivity, be lightweight but with high mechanical strength, have low corrosion impact and low moisture uptake, and good fire behavior properties. Over the years, a wide range of natural and synthetic materials have been used, including straw, wool, cork, mineral wool, expanded polystyrene beads, polyurethane foams, and phenolic foams. The latter has attracted much attention due to its excellent properties, including low thermal conductivity, flame retardancy, low flammability, and low smoke emission during combustion [1,2]. Phenolic foam installed in interior and exterior building walls and roofs, air crafts, and marine vessels can significantly reduce the heat transmission and also provides some protection from fire accidents [3,4]. Moreover, its low price and high chemical resistance compared to other insulation foams or materials help to diversify its application areas [5].

Despite the many advantages of phenolic foams, their relatively low mechanical strength limits their application in many areas. To address this shortcoming, the mechanical properties of the foam have been improved either by chemical modification or by the incorporation of various additives [1]. Chemical modification is achieved by introducing long flexible chains into the structure of the phenolic resin via chemical reaction. In the physical toughening method, organic or inorganic materials, fibers, and glass are blended into the phenolic resin prior to curing. Our recent paper compares the toughening effect of various additives such as urea, nano clay, sodium silicate, and lignin on phenolic foam [6]. The chemical modification method is preferred to physical toughening as it tends to provide better improvements in mechanical properties. Synthetic polymers such as polyurethane [7], polyethylene glycol [8], and polyether [9] have been used to toughen phenolic foams via the chemical modification method. Biomass-derived raw materials such as lignin, tannin, and cardanol have also been employed to physically and chemically toughen phenolic foam [10]. Most of the biomass-derived materials contain phenolic groups in their structure which can react with the functional groups present in phenolic resin. The incorporation of such materials into the synthesis enables the reduction in the amount of phenol used. Hence, toughening with biomass-derived materials not only improves the mechanical properties, but also eases the dependence on petroleum-derived feed stock. Phenolic foams are industrially produced from fossil-feedstock-derived phenol and formaldehyde [11]. Current concerns regarding the environment, oil price hike, and fossil fuel scarcity have driven researchers and manufacturers to shift their focus towards more sustainable alternative feedstocks. Biomass represents a sustainable option for producing chemicals and fuels due to its ease of availability, abundance, and low cost [12]. During the past decade, tremendous research has been published on technologies and methods for converting biomass into chemicals and biofuels [13,14]. Several more sustainable phenolic foams have been produced recently by replacing phenol and formaldehyde with biomass-derived materials such as tannin [15,16], cardanol [17], lignin [18], bio-oil [19], glyoxal, furfural, and hydroxymethyl furfural. 

Bio-oils produced from various biomass sources have already been used in the synthesis of phenolic resin and foam [19,20,21]. Bio-oils produced during the pyrolysis of biomass contain phenol, cresols, guaiacol, desaspidinol, catechol, resorcinol, ketones, aldehydes, and many other phenolic compounds with long unsaturated alkane chains [22,23]. They are well suited for chemical production and, due to their high phenol content, are also appropriate for replacing phenol in phenolic resin production, either partially or completely. The long flexible chains present in the bio-oil-derived phenols could impart toughness to the phenolic foam. Bio-oil extracted from whole tree feedstock has been utilized to prepare phenolic resin. Hence, it could help ease the dependence on nonrenewable petroleum resources. 

Various types of bio-oils derived from different biomass feed stocks have been employed to prepare phenolic resin and foam. Yu et al. demonstrated a 10.5–47.4% increase in compressive strength of phenolic foams with 10–20% bio-oil substitution [22]. Thus, bio-oils are also able to enhance the mechanical properties of the foam without the need for additional toughening agents. Chaouch et al. demonstrated that 50% bio-oil substituted resin showed better properties than conventional phenolic foam and the particle board prepared using this resin displayed better internal bond (IB) strength [21]. Foams prepared from 50% white birch bark-derived bio-oil substituted phenolic resin showed higher compressive strength and elastic modulus than traditional phenolic foam [24]. Tung oil substituted foam exhibited a 69–87% increase in mechanical strength with only a 6% substitution rate. The long and flexible chains in the tung oil were responsible for toughening the phenolic foam and, as a result, increasing its mechanical strength [25]. 

In this work, we utilized bio-oil extracted from date palm waste for partial substitution of phenol in phenolic resin synthesis and corresponding foam production. The impact of date palm bio-oil on the mechanical and thermal properties of the foam was also evaluated. Date palm trees are usually grown in tropical and dry regions, especially in the Middle East and the North African (MENA) countries. Around 62% of the date palm trees are located in those countries and the remainder is spread between India, Pakistan, the United States, and the Canary Islands [26]. Date palm trees are cultivated as crops as their fruit, known as dates, have a wide range of applications in the food processing industry [27]. They are rich in nutrients and are usually consumed as fruit and also used in making cookies, cakes, and syrups [28]. Every year, after fruit harvesting, a huge amount of waste is produced and only a small percentage of it is utilized for making rope, baskets, fish traps, furniture, and boats. A major portion of the waste is used for generating steam in boilers, burnt, or sent to landfill. Several studies have been reported recently on utilizing date palm waste for the production of biofuels [29], biochar [30], and activated carbon adsorbents. As the date palm tree contains lignin, hemicellulose, and cellulose in its structure, it could be a potential precursor for bio-oil production. Depletion of fossil fuels and the oil price hike have driven researchers to develop more sustainable alternate energy sources. Recently, there has been an exponential increase in research focusing on methods to convert biomass to energy. 

Date palm waste can be converted to bio-oil via the fast pyrolysis method, which could be used for energy production [29,31,32,33]. In this study, the potential of biomass-derived date palm bio-oil as partial substitute for phenol in the synthesis of phenolic resin and foam was examined. In addition, the effects of the date palm bio-oil on key phenolic foam properties, especially thermal stability and compressive strength, were evaluated. Makkawi et al. [34] detailed the characterization of feedstock and the products obtained from the pyrolysis of date palm waste. The major components in the date palm bio-oil are various kinds of phenols, D-Allose, catechol, and apocynin [23], with the phenols reaching nearly 50% of the total bio-oil [34]. In this study, date palm (DP) bio-oil substituted resin was synthesized by replacing 10%, 15%, and 20% of phenol with date palm bio-oil and the properties of the resin were analyzed. Foams from all the substituted resin were produced by a standard foaming method. The density, thermal conductivity, mechanical properties, and thermal stability of all the foams were analyzed and compared with those of the traditional phenolic foam. 

## 2. Experimental

### 2.1. Materials

Date palm waste was collected from trees within the vicinity of the American University of Sharjah, UAE. The waste consisted of equal fraction of leaves, leaf stems, and empty fruit bunches. Phenol (detached crystal of 99% purity) was obtained from Fisher Scientific, Loughborough, UK. Formaldehyde (37%), sodium hydroxide, and n-hexane were supplied by Merck, Damstadt, Germany. The catalyst methane sulfonic acid (Lutropur MSA) and the ethoxylate castor oil surfactant (Agnique CSO-30) used in the foaming process were supplied by BASF, Ludwigshafen, Germany. 

### 2.2. Preparation of the Date Palm Bio-Oil

The date palm bio-oil was produced from date palm waste via pyrolysis method using an auger reactor at 525 °C. The date palm waste feedstock preparation methods, reactor description and operation conditions, pyrolysis procedures, and product analysis results are detailed in a recent publication [34]. The chemical composition of the DP bio-oil used in this study was analyzed via GC–MS using undecane as an internal standard. The major components present in the DP bio-oil include monosaccharides, hydrocarbons, phenols, alcohols, and ketones. Makkawi et al. reported a detailed characterization of the date palm bio-oil obtained from the date palm waste in recent publications [23,34].

### 2.3. Synthesis of Phenolic Resin (PR)

Resol-type phenolic resin was synthesized by the standard condensation process between phenol (P) and formaldehyde (F) in the presence of sodium hydroxide with an F/P molar ratio of 1.5. Initially, crystalline phenol (1 mol) was charged into a three-neck round-bottomed (RB) flask maintained in an oil bath equipped with a magnetic stirrer. Sodium hydroxide aqueous solution (15 mL, 50%, *w*/*v*) was added to the reaction mixture to adjust the pH to 9 and the reaction was maintained for 30 min at 40–45 °C. Then, 60% of the required formaldehyde was added dropwise into the reaction mixture while maintaining the temperature at 60 °C and this was reacted for 1 h. NaOH solution (50%, 5 mL) was added prior to the addition of formaldehyde and the temperature was increased to 85 °C. The remaining 40% of the formaldehyde was added to the reaction when the temperature reached 70 °C. The reaction was continued for another 2.5 h at 85 °C, after which the resin was cooled and distilled to obtain the required viscosity.

### 2.4. Synthesis of Date Palm Bio-Oil Substituted Phenolic Resin (DPR)

**Figure 1:**
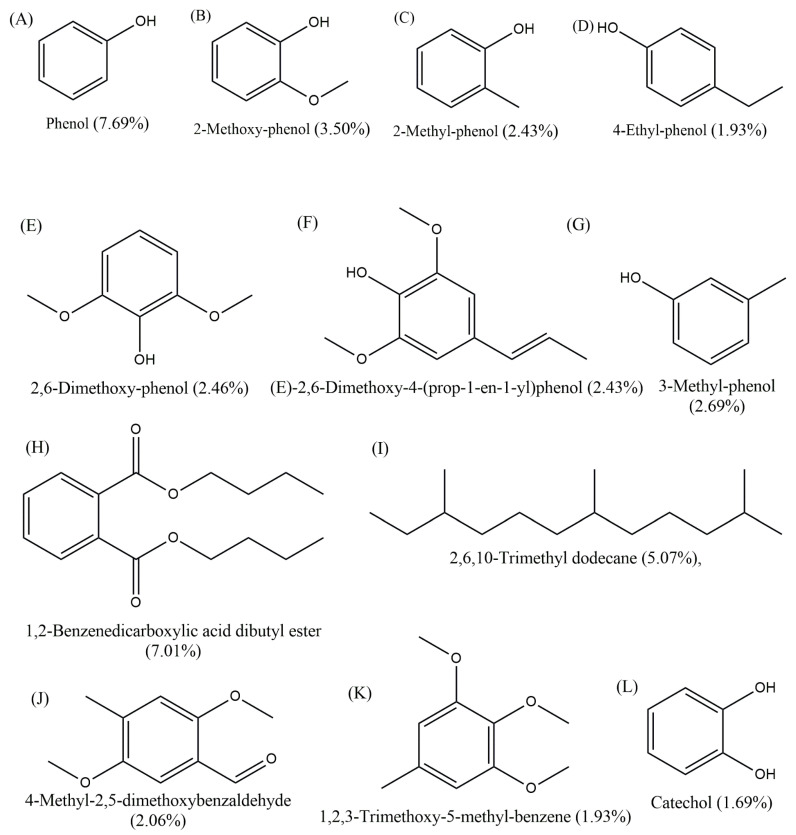
Chemical structures of the major compounds present in the date palm bio-oil.

The synthesis of date palm bio-oil substituted resin was similar to the synthesis of standard phenolic resin. Date palm-bio-oil substituted phenolic resin was prepared by replacing 10, 15, and 20 wt.% phenol with DP bio-oil. The phenol percentage in date palm bio-oil is less than the phenol obtained from petroleum resource. Hence, the F/P ratio in the bio-oil substituted resin will be lower than the pure PR resin. The ratio will increase with percentage of substitution. Major phenols present in the DP bio-oil (Figure 1) were reported in recent publication [23,34]. Date palm bio-oil obtained via pyrolysis was used directly in the substituted resin synthesis. In the date palm bio-oil substituted resin, various percentages (10, 15, and 20 wt.%) of bio-oil dissolved in ethanol were added along with the phenol. The remaining reaction steps were similar to those explained in the synthesis of pure phenolic resin. The resultant resins are denoted as 10DPR, 15DPR, and 20DPR, respectively. The physical properties such as viscosity, solid content, pH, and free formaldehyde content (ISO 9397 [35]) of all the resins were analyzed. 

### 2.5. Resin Characterization

The resin viscosity was measured using NDJ-8S Rotational Viscometer (Shanghai, China) at room temperature (25 °C). The solid content of the resin was analyzed by the method described in ASTM D4426-01 [36]. pH of both traditional and substituted resins was measured by digital pH meter (Bluelab Combo Meter, Sacramento CA, USA). Free formaldehyde content in all the synthesized resins was measured via the hydroxylamine hydrochloride method in accordance with ISO 9397 [35]. 

### 2.6. Synthesis of Date Palm Bio-Oil Substituted Phenolic Foam (DPF)

Phenolic foam and date palm bio-oil substituted foams were prepared via a similar procedure. Initially, resin and surfactant (Agnique CSO 30) were mixed with a hand blender. Hexane, the blowing agent, was added to this mixture and mixed for one minute. The catalyst, methane sulfonic acid, was added immediately and mixed again using the hand blender. The reaction mixture was immediately poured into a closed-type mold and cured at 80 °C for an hour. Post-curing was performed at 60 °C for 2 h. Compositions of the prepared formulations are shown in PF, 10DPF, 15DPF, and 20DPF, which denote traditional phenolic foam, foam from 10% date palm bio-oil substituted resin, 15% date palm bio-oil substituted resin, and 20% date palm bio-oil substituted resin, respectively.

### 2.7. Foam Characterization

The apparent densities of all foam samples were measured as per the ASTM D1622 standard [37]. The thermal conductivity of both PF and DPF foams was analyzed using a Tci thermal conductivity analyzer (C-Therm, Fredericton, New Brunswick, Canada) at room temperature (25 °C). Morphology of the foams was examined using a scanning electron microscope (TESCAN, Brno—Kohoutovice, Czech Republic) with an accelerating voltage of 20 kV. Foam samples were first sputter coated with a gold conductive layer and then visualized under SEM. The mean cell size, cell size distributions, and cell wall thickness of each foam were calculated from at least 150 cells on the SEM images using ImageJ software (https://imagej.net/ij/download.html, accessed on 21 June 2023). The thermal stability of the foam samples was determined via thermogravimetric analysis (Perkin Elmer, Hopkinton, MA, USA) at a scan rate of 10 °C min^−1^ under nitrogen (20 mL/min) from 30 to 850 °C. The compressive strength of the pure and toughened foams was measured using a universal testing machine (Instron, Norwood, MA, USA) at room temperature according to ASTM D1621 [38]. All results were reported as an average of three samples each with a size of 30 × 30 × 30 mm^3^. 

## 3. Results and Discussion

### 3.1. Characterization of Date Palm Bio-Oil, PR and DPR

In this work, date palm bio-oil derived from date palm waste was used for substituting phenol in phenolic resin production. Phenols obtained from petroleum resources are highly pure and simple chemical processes and purification methods are required for their production. On the other hand, bio-oil chemical combinations are highly complex; as a result, careful structural analysis is required for their application as a raw material for chemical synthesis. GC–MS analysis is usually used to quantify the chemical compounds present in bio-oils. The GC–MS analysis confirmed the presence of a major 30 compounds, representing 75% of the DP bio-oil including various phenols such as phenol (7.69%), 2-methoxy-phenol (3.50%), 2-methyl-phenol (2.43%), 4-ethyl-phenol (1.93%), 2,6-dimethoxy-phenol (2.46%), (E)-2,6-dimethoxy-4-(prop-1-en-1-yl)phenol (2.43%), and 3-methyl-phenol (2.69%). Other significant compounds are various alcohols, aldehydes, esters, alkanes, and ketones, including 1,2-benzenedicarboxylic acid dibutyl ester (7.01%), 2,6,10-trimethyl dodecane (5.07%), 4-methyl-2,5-dimethoxybenzaldehyde (2.06%), 1,2,3-trimethoxy-5-methyl-benzene (1.93%), and catechol (1.69%). Chemical structures of the major compounds in the DP oil are shown in Figure 1. 

**Figure 2:**
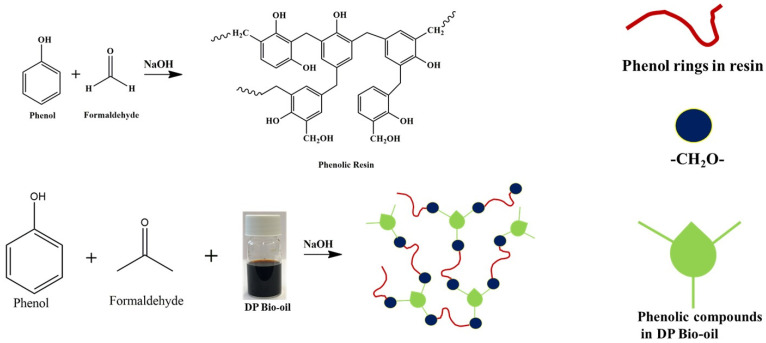
Schematic representation of the mechanism of date palm bio-oil substituted resin.

Date palm bio-oil substituted phenolic resins were prepared by substituting phenol with various percentages of bio-oil. The phenolic compounds present in the DP bio-oil could react with the formaldehyde molecule, and the long chains of the DP bio-oil were introduced into the resin network. A schematic representation of the reaction mechanism is shown in Figure 2.

Table 1 presents the properties of the standard phenolic resin and the date palm bio-oil substituted resins. It has been shown previously that long alkyl chains in the phenols present in the bio-oil and also the other hydrocarbons, ketones, and aldehydes could provide a toughening effect to the resin by introducing chain flexibility [39]. 

As shown in Table 1, the viscosities of 10DPR and 15DPR are both lower than that of the standard phenolic resin (PR), while the viscosity was found to increase with increasing percentage of bio-oil substitution. In PF, the F/P ratio was 1.5 and the reactivity of phenols obtained from the petroleum sources were higher than those from the biomass. In addition, the free formaldehyde content in PR resin is very low compared to all other substituted foams, which also confirms the high reactivity of phenols in the PR resin. In DPRs, the percentage of phenols available for cross-linking was lower and in the order of 10DPR < 15DPR < 20DPR, hence the F/P ratios in substituted resins were lower than that in PR, which also results in the lower viscosities of 10DPR and 15DPR. Also, the steric hindrance due to the complexity of the DP bio-oil and the presence of several other molecules or functional groups reduces their reactivity with formaldehyde. Bio-oil contains a significant percentage of water content, which is also a reason for the low viscosity of the substituted resin. The viscosity of the substituted resin increased with an increase in date palm bio-oil percentage. This is due to the greater availability of functional groups (phenols) for condensation reaction and also the steric hindrance from the long alkyl chains of hydrocarbons in the bio-oil. The increase in F/P ratio with respect to the percentage of bio-oil substitution also helps to enhance their viscosity. The solid contents of 10DPR and 15DPR are lower than that of pure phenolic resin due to their low viscosity and the high water content of the DP bio-oil, whereas the resin 20DPR shows a similar solid content to PR resin and is higher than the other DP bio-oil substituted resins. The lowest solid content in 15% DP bio-oil substituted resin might have been caused by the excessive percentage of phenol in the oil which was not consumed with formaldehyde in the reaction due to the steric hindrance. During the solid content analysis, the unreacted phenol evaporated and this led to a lower solid content. Furthermore, the free formaldehyde content in 15DPR is significantly higher than that in PR and DPR, which also confirms the presence of unreacted phenol in the resin. The high solid content in 20DPR is due to the high viscosity of the resin and increased percentage of DP bio-oil in the resin. The high F/P ratio due to 20% substitution and the presence of long alkyl chains enhances the resin viscosity. The PR and 20DPR have almost similar solid content even though the viscosity of 20DPR is significantly high. This is due to the water content present in DP bio-oil and also the lower reactivity of the phenols present in the DP bio-oil. The high viscosity of 20DPR is mainly due to the highest percentage of high-molecular-weight complex phenols present in the resin. Due to the steric hindrance from the complex molecules in bio-oil, there is a high amount of free phenols (petroleum based) present in the 20DPR resin. These phenols will evaporate during the solid content analysis, causing a lower solid content than the expected high value with respect to its viscosity. Free formaldehyde in the pure phenolic resin is very low (0.17%). However, the substituted resin shows relatively high formaldehyde content and it increases in proportion to the date palm oil percentage. This also suggests that the lower reactivity of date palm bio-oil resin is due to the steric hindrance from its structure. Substituted resin has lower pH than traditional phenolic resin due to the acidity of the phenols in the bio-oil. 

### 3.2. Characterization of PF and DPF

**Figure 3:**
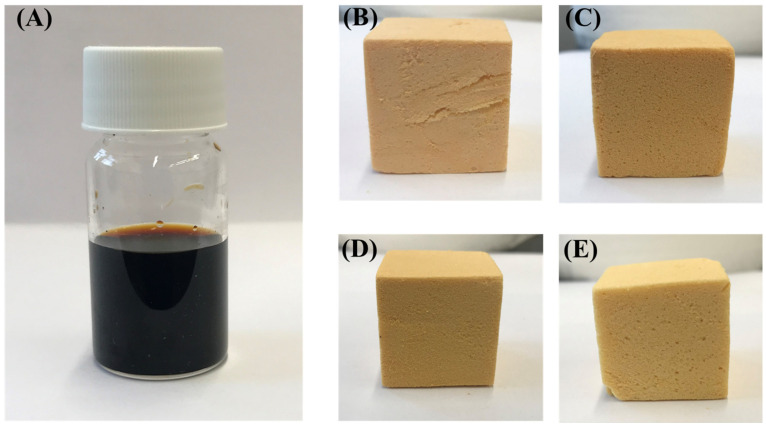
Images of (**A**) date palm (DP) bio-oil, (**B**) standard phenolic foam (PF), (**C**) date palm bio-oil substituted foams (10DPF), (**D**) 15DPF, and (**E**) 20DPF.

Foams were formulated from both phenolic and date palm bio-oil substituted resin. Several foams were formulated by changing the concentrations of Agnique CSO-30, hexane, and MSA. It was found that the properties of the foams could easily be modified by changing the concentration of surfactants, blowing agents, and catalysts. Table 2 presents selected formulations of PF and DPF foams and Figure 3 shows images of the date palm bio-oil, PF, and various DPF samples.

#### 3.2.1. FT-IR Characterization of PF and DPF

**Figure 4:**
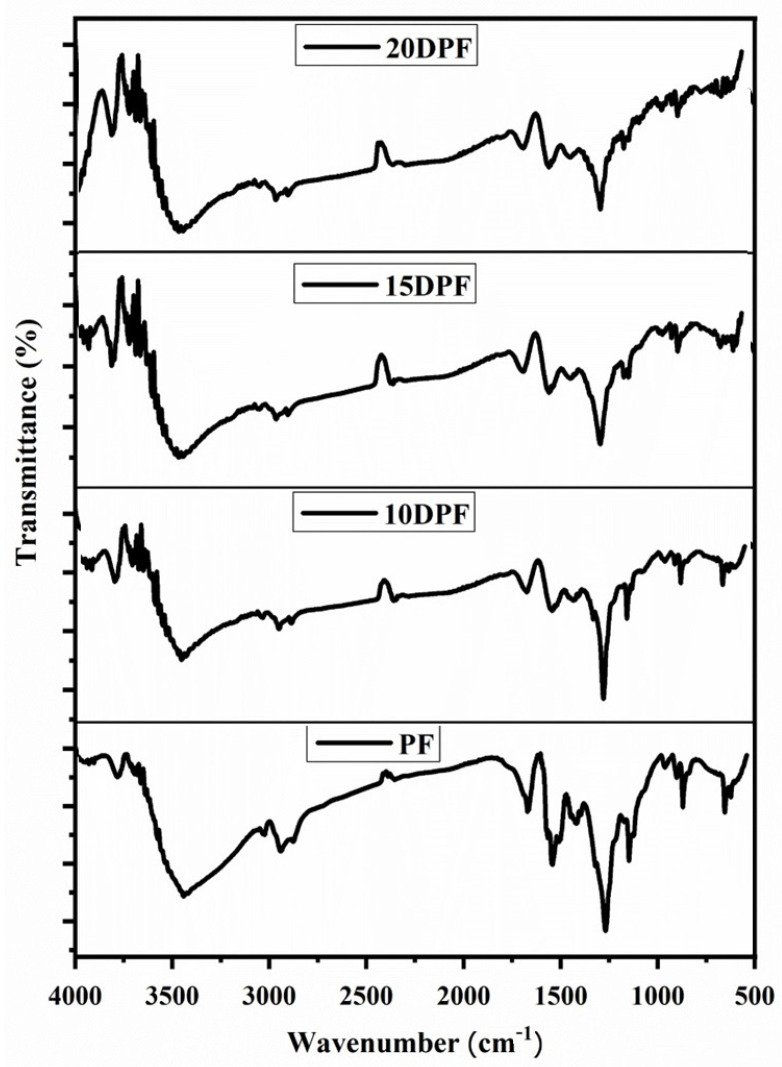
FT-IR spectra of date palm bio-oil substituted foams 20DPF, 15DPF, and 10DPF and standard phenolic foam (PF).

The FT-IR spectra of PF and various DPF samples are shown in Figure 4. The broad peak at 3340 cm^−1^ in the PF spectra corresponds to the -OH hydroxyl group. Peaks at 1605 cm^−1^ and 1498 cm^−1^ represents the -C-C aromatic ring stretch and aliphatic -CH_2_ bend, respectively. The phenolic -C-O stretching peaks are seen at 1199 cm^−1^. The broad hydroxyl peaks of the PF foams are split into two peaks in all the bio-oil substituted foams. The number of phenolic groups in date palm oil is less and it is sterically hindered as compared to the pure phenolic foam. However, the -CH stretching vibrations in the range of 1604 cm^−1^ of the aromatic rings are retained in all bio-oil substituted foams. All the substituted phenolic foam samples show similar peaks in the region between 1650–1000 cm^−1^. Bio-oils also contain the strong stretching vibrations of -C-O groups of the aromatic rings, as observed in the PF foams. 

#### 3.2.2. Apparent Density of PF and DPF

Physical properties of traditional PF foam and the bio-oil substituted foams with various percentage of date palm bio-oil are listed in Table 3.

**Figure 5:**
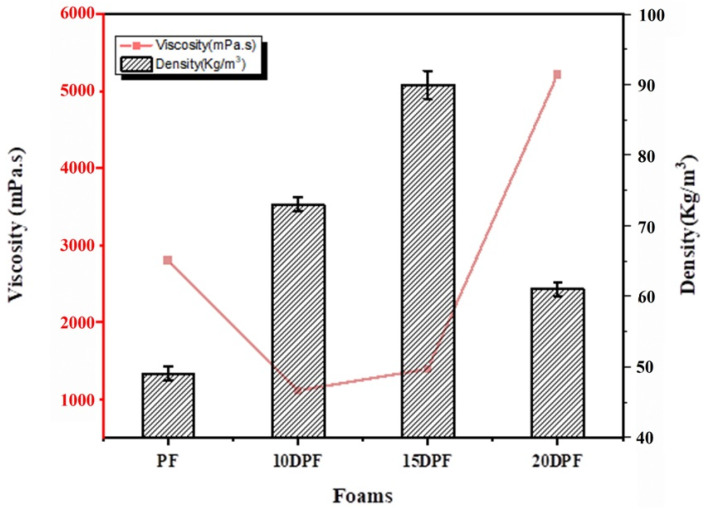
The densities and viscosities of the PF and DPF samples.

Apparent density of phenolic foams is highly dependent on the viscosity of the resin from which it was formulated [40], the cross-linking efficiency of the reactive functional groups present in the resin, and the amount of blowing agents in the resin foaming mix. The traditional phenolic foam has a density of 48 kg/m^3^, whereas the DPF samples have higher densities. PF foam density increased with 10% and 15% bio-oil substitution, whereas it showed a reduction with 20% substituted resin, but still higher than pure PF. In PF, phenols are highly reactive; hence, the cross-linking reaction during the foaming process is facile. In low-viscosity resin, the mixing of foaming agents is easy and the expansion rate during the foaming process is also high, which will result in a foam with a low density. In 10DPF, the low viscosity of the resin facilitates the uniform distribution of surfactants, blowing agents, and catalyst in the resin and also helps the cross-linking reaction between the larger number of reactive sites present in the bio-oil and the resin. Low-viscosity resin usually results in a low-density foam. But here, though the viscosity of 10DPR is less than PF, the foam formulated from it shows a higher density. This is due to the steric hindrance offered by the long alkyl chains in the DP bio-oil. The long chains trap the blowing agents inside the chains and prevent them from expansion. In 15PDF, density is increased to 90 kg/m^3^, which is even higher than that of 10DPF due to the slightly higher viscosity and more steric hindrance of the alkyl chains of the bio-oil. Since the viscosity of 20DPR is higher than all substituted resin and also the PR, it is expected to have the highest density. However, the foam exhibits a density of 61 kg/m^3^, which is lower than DPF and higher than pure PF. This unusual behavior of the 20DPF is explained by the presence of high water content in the resin originating from the high content of DP bio-oil and elevated level of free formaldehyde in the resin due to the low reactivity of the bio-oil phenols. This water acts as a nucleating agent along with the blowing agent, which resulted in expansion of the resin mixture and, hence, a low-density foam. The densities of PF and DPFs and their corresponding resin viscosity are shown in Figure 5.

#### 3.2.3. Thermal Conductivity PF and DPF

Thermal conductivity is an important physical property that is the primary factor in determining the thermal insulation efficiency of a foam. Foams with low thermal conductivity are preferred for insulation due to their improved ability to reduce energy loss while minimizing the insulation thickness required. The thermal conductivity of phenolic foam was found to increase after date palm bio-oil substitution. Pure PF has a thermal conductivity of 0.035 W/m·K, which increased to 0.036–0.040 W/m·K after DP bio-oil substitution. Analysis of variance (ANOVA) was conducted to test the statistical significance of the thermal conductivity results by comparing between groups for foams with different DP bio-oil content. The results indicate a significant difference at the *p* < 0.05 level for the four groups, *F*(3,8) = 10.3, *p* = 4.08 × 10^−3^.

**Figure 6:**
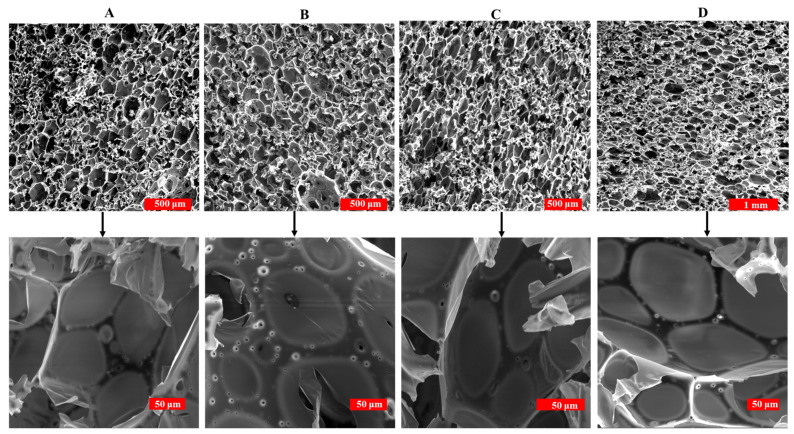
SEM images of the standard PF (**A**), 10DPF (**B**), 15DPF (**C**), and 20DPF (**D**).

The high density contributes to an increase in the thermal conductivity of the 10DPF and 15DPF. Li et al. also observed a similar increase in thermal conductivity with density of the bark-derived bio-oil substituted foam [24]. In foams, thermal conductivity through gas is the most predominant component in the total heat transferred as the foams contain a huge volume of gas trapped inside their structure. The conduction through solid is more prevalent in highly dense foams [41]. Though dense foams show high thermal conductivity, it is not directly proportional to the density. Other factors such mean cell size, closed cell content, and voids on the cell walls also contribute towards the thermal conductivity [42]. Though the density of 10DPF is lower than 15DPF, it shows a higher conductivity, of 0.040 W/m·K, than the conductivity value of 0.038 W/m·K in 15DPF. The large number of voids present on the cell walls of the 10DPF (Figure 6B) explains the high conductivity of 10DPF. The gas entrapped inside the cells might escape through the voids, leading to an increase in the conductivity of 10DPF. Among all the three substituted foams, 20DPF shows similar thermal conductivity to that of PF due to a similarity in the cell structure, cell size distribution, and low density. 

#### 3.2.4. Morphology of PF and DPF

The microstructure of the PF and bio-oil substituted foams was analyzed using a scanning electron microscope (SEM). The cell structure plays a crucial role in determining the properties of insulation foam, especially the thermal conductivity and mechanical strength. In order to have a low thermal conductivity and high mechanical strength, uniform, small, and closed cells are required. The SEM images of PF and all DPF samples are shown in Figure 6 and their cell properties are listed in Table 4. 

**Figure 7:**
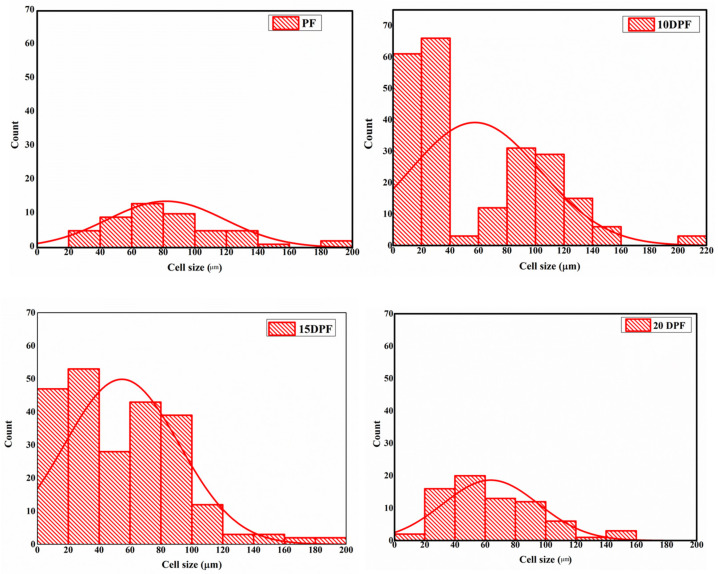
Cell size distributions of traditional phenolic foam (PF) and date palm bio-oil substituted foam (DPF).

All the foams have closed cells, with some perforations on cell walls due to the evaporation of water present in the corresponding resin. Traditional PF foams have oval-shaped closed cells with a mean diameter of 85.9 ± 34 μm and a cell wall thickness of 12.63 ± 7 μm, as shown in Table 4. The cell sizes in PF foams range from 25 to 188 μm, as shown in Figure 7. Perforations are seen in all the DPF foams as the water content in the date palm resin is higher than in standard phenolic resin. Prior to curing, DPF and PF are aqueous compatible, but during the foaming and curing process, they are converted to a cross-linked water incompatible foam. The transition from water compatibility to noncompatible state during the curing results in the expulsion of water [43]. This expelled water causes perforations in the cell walls [44,45]. Since the DPR contains more water content, the expelled water during the cross-linking process will be higher, which leads to larger number of perforations on the foam. Since the viscosity of the 10DPR is lower than the PR, it is expected to have a lower density with large cells. Due to the steric hindrance from the long alkyl chains present in the bio-oil, bubble formation and expansion become restricted, which leads to a high density with small cells. The mean cell size was decreased to 57.09 ± 45 μm, with thick cell walls having thicknesses ranging from 9.4 to 21.68 ± 7 μm. The high solid density of the 10DPF was concentrated in between the cell walls and strut region. Among all the substituted foams, 15DPF shows the most uniform cell structure, with long and elongated cells having a mean cell diameter of 54.4 ± 36 μm. The high viscosity of 15DPR compared to PR and 10DPR imposes a restriction on the foam expansion, which results in a dense foam with small and elongated cells. The long alkyl chains in the resin also inflict restriction on the foam expansion. In 20DPF, due to the high viscosity of the corresponding resin, the cross-linking reaction was poor, which leads to a nonuniform foam expansion and a reduction in density of the resulting foam. As a result, the cell structure becomes irregular, with cell size ranging from 18 to 150 μm and a mean cell size of 64.5 ± 32 μm. The 10DPF and 20DPF have cells with almost similar size and cell wall thickness due to their almost close density. All the bio-oil substituted foams have thicker cell walls than the traditional phenolic foam due to their high density and small mean cell size. The steric hindrance offered by the long alkyl chains in the bio-oil also contributes to the increase in the cell wall thickness in substituted foams. 

#### 3.2.5. Mechanical Properties of PF and DPF

The mechanical performance of the bio-oil substituted foams was analyzed by measuring the compressive strength on a universal testing machine. The standard PF exhibited a compressive strength of 108 kPa. Apparent density and the cell structure of the foam play a decisive role in determining the compressive strength [46]. Foams with high density and thick cell walls tend to have high compressive strength which increases proportionally with density. Li et al. found that the outer-bark-derived bio-oil substituted phenolic foams with high density exhibit higher compressive strength than inner-bark-derived oil substituted foams [24]. The differences observed in the compressive strength of the bark-derived oil substituted phenolic foams were due to differences in density. PF shows a density of 49 kg/m^3^ and a cell wall thickness of 12.6 ± 7 μm. Analysis of variance (ANOVA) was conducted to test the statistical significance of the compressive strength results by comparing between groups for foams with different DP bio-oil content. The results indicate a significant difference at the *p* < 0.05 level for the four groups, *F*(3,8) = 478, *p* = 2.34 × 10^−9^.

**Figure 8:**
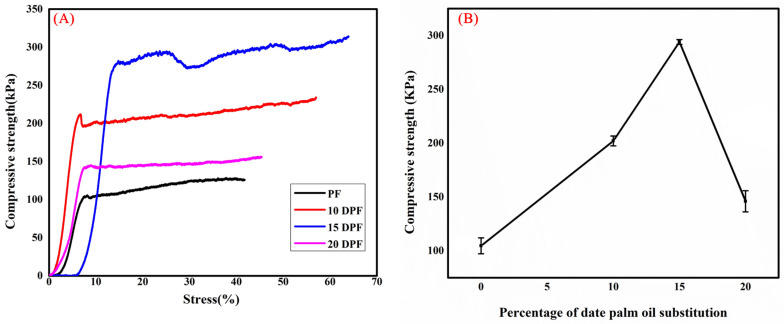
Stress–strain curves of phenolic (PF) and date palm bio-oil substituted foam (DPF) (**A**). Changes in compressive strength of the foam with increase in bio-oil substitution (**B**).

The compressive strength of the traditional PF was lower due to its low apparent density and thin cell walls. Compressive strength of DP bio-oil substituted foams was found to be higher than that of standard PF. As the percentage of substitution was increased, the compressive strength also increased until an optimum percentage, after which the strength started to deteriorate (Figure 8B). Yu et al. also observed similar trend in the compressive strength of the phenolic foam modified with bio-oil [22]. They found that the compressive strength of the phenolic foam increased with up to 20% bio-oil substitution, while further incremental increases weakened the compressive strength due to cell collapse. In this study, the 10DPF and 15DPF show compressive strengths of 202 kPa and 294 kPa, respectively. The enhancement in compressive strength is caused not only by the increased density of the foam but also by the presence of long alkyl chains of various moieties in the date palm bio-oil. The long chains offer greater resistance to rupture/fracture under applied stress by absorbing the destruction energy, controlling the rate and propagation of induced cracks, and, thereby, slowing the cell damage during compression. Hu et al. observed that the long alkyl chains present in the lignosulfate contribute to the toughness to a greater extent than the pure benzene ring in the PF [47]. Hence, the lignosulfonate-added phenolic foam exhibits higher compressive strength than pure phenolic foam. In addition to alkyl chains, all the substituted foams have thick cell walls as compared to standard PF. Thick cell walls absorb compression stress, effectively distribute the structure throughout the foam, and increase the compression resistance [15,41]. The 20DPF exhibited the lowest compressive strength of 146 kPa among all the substituted foams due to its low density of 61 kg/m^3^. The stress–strain curves of all the foams are shown in Figure 8A. 

#### 3.2.6. Thermal Properties of PF and DPF

**Figure 9:**
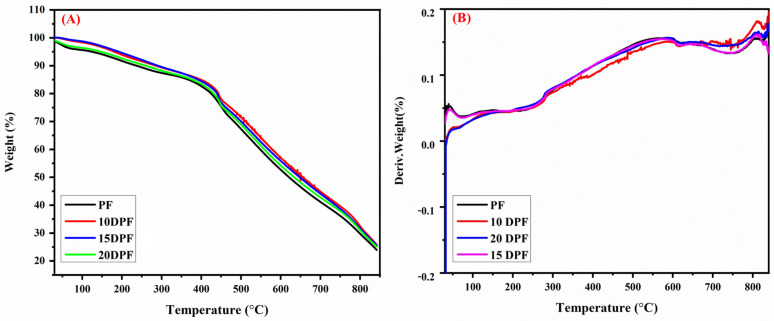
(**A**) TGA and (**B**) DTG curves of pure traditional phenolic foam (PF) and date palm bio-oil substituted (10DPF, 15DPF, and 20DPF) phenolic foam (DPF).

Thermal stability of the foams can be analyzed via TGA studies. The TGA and DTG (derivative thermogram) plots of standard PF and the substituted foams are shown in Figure 9. Sometimes, thermal stability of the foams is adversely affected by addition of toughening agents [20]. On the other hand, some toughened foams have enhanced the thermal stability compared with the standard PF [48]. Hence, it is important to study the effect of date palm bio-oil on the thermal resistance of phenolic foam. All the DP bio-oil substituted foams show better thermal stability and residual weight percentage than the standard PF foams over all the studied temperatures. Thermal stability of other bio-oil substituted foams has been reported to decrease considerably [20] but the date palm bio-oil substitution does not appear to affect the thermal stability of the foam. The thermograms of PF and DPF were divided into four regions: (a) below 100 °C, (b) between 100 and 200 °C, (c) between 200 and 600 °C, and (d) above 600 °C. Thermal degradation curves of phenolic foam have three main regions. The first region corresponds to the evaporation of water and small molecules such as unreacted phenol, formaldehyde, and blowing agent. In the second region, dehydration due to further curing of the resin may occur [49]. Also, decomposition of surfactant and curing agents happens in this region. Major weight loss occurs in the third stage as it denotes the ether bond cleavage, or the methylol group dehydrogenation on the aromatic ring [50], hydroxyl radical formation from the phenolic hydroxyl group [51], and its reactions with methylene and hydroxymethyl groups. The region above 600 °C denotes the degradation of long alkyl chains and methylene bridges [52]. 

The decomposition data of pure PF and DPF are listed in Table 5. The initial decomposition temperature, T_−5%_ (the temperature at which 5% mass loss occurs), for each of the toughened foams is higher than that of pure PF. The values shown in Table 5 clearly demonstrate the thermal stability of date palm bio-oil toughened phenolic foam. In PF, the initial weight loss occurs at around 125 °C (T_−5%_), whereas in DPFs it occurs in the range between 150 and 200 °C. In 10DPF and 15DPF, the degradation temperature is increasing with the percentage of DP bio-oil, while in 20DPF, T_−5%_ occurs at a lower temperature, which is even higher than pure PF. The high thermal stability of DPFs at low temperature is due to the thick cell walls as compared to the PF. The thick walls prevent the loss of low volatiles at low temperature [19]. In 20DPF, the T_−5%_ occurs at a lower temperature due to its low density and the presence of higher number of alkyl chains. The thermal stability of alkyl chains is less than benzene rings [20]. Observation of the residual weight percentage at 200 °C also supports the high stability of DPF, as these show higher residual weight than pure PF. Density also plays a role in the thermal stability of DPF foam. At T_−5%_, the thermal stability of DPF follows the order of 15DPF > 10DPF > 20DPF > PF, a density-dependent degradation pattern. Phenolic foams with high density show better thermal stability [46]. Like in PF, the maximum weight loss occurs in the region between 500 and 600 °C and the T_max_ of all the foams are listed in Table 5. The weight loss in this region is mainly due to the ether linkages and the alkane chains [53]. The T_max_ of 10DPF and 15DPF are closer and the 20DPF shows maximum degradation at 568 °C, which is higher than pure PF. The residual weight percentage at 400 °C, 600 °C, and 800 °C is in the order of 10DPF > 15DPF > 20DPF > PF. At higher temperatures, 10DPF shows higher thermal stability than 15DPF and 20DPF due to the presence of fewer alkyl chains in them than other substituted foams. The alkyl chains introduced into the phenolic matrix smoothen the resin chain motion, which facilitates quick removal of volatile compounds [20]. The 15DPF and 20DPF contain more alkyl chains than 10DPF, which degrades faster; hence, it shows less stability than 10DPF. Apart from the alkyl chains, the high density of 15DPF also facilitates higher thermal stability than 20DPF. 

## 4. Conclusions

In this work, the potential of bio-oil derived from date palm waste as a substitute for phenol in phenolic resin production and its toughening impact on the foam properties were studied. Resins of up to 20% DP bio-oil substitution were prepared and used for foam production. The long flexible chains as well as various functional groups on the date palm bio-oil provide faster cross-linking than pure phenolic resin. SEM images of the foam cell structure prove the impact of date palm bio-oil on the cell size, distribution, and cell wall thickness. All the substituted foams exhibited a uniform structure with closed cells and thick cell walls compared to the pure phenolic foam. Positive improvements in the cell structure also explain the enhancement in compressive strength. Though thermal conductivity of 10% and 15% substituted foams increased, we could produce a foam with similar thermal conductivity and high compressive strength with 20% bio-oil substitution. Specifically, the compressive strength increased to 93%, 181%, and 40% with 10%, 15%, and 20% DP bio-oil substitution. Hence, the mechanical properties of the resulting foams were improved without the need for any additional toughening agents. Several bio-based toughening agents decrease the thermal stability of the foam while increasing its mechanical strength. However, the date palm bio-oil was found to increase the mechanical strength while also maintaining thermal stability. Furthermore, the use of material from date palm waste can help to reduce the amount of waste going to landfills. Therefore, the date palm bio-oil substituted phenolic foams have improved environmental profile and superior mechanical properties compared to traditional phenolic foams.

## Figures and Tables

**Table 1:** Properties of traditional phenolic resin (PR) and date palm bio-oil substituted phenolic resin (DPR).

Resin	Viscosity @ 25 °C (mPa·s)	Solid Content (%)	pH	Free Formaldehyde Content (%)
PR	2800	75.9 ± 0.4	10	0.178
10DPR	1110	69.8 ± 0.3	9.7	0.470
15DPR	1390	67.9 ± 0.4	9.6	0.715
20DPR	5210	75.8 ± 0.2	9.6	1.18

**Table 2:** Formulations of phenolic (PF) and date palm bio-oil substituted foams (DPF).

	PF	10DPF	15DPF	20DPF
Resin viscosity (mPa·s)	2800	1110	1390	5210
Resin (g)	25	25	25	25
Agnique CSO30 (g)	1	1	1	1
Hexane (mL)	4	4	4	4
L-MSA (mL)	2	2	2	2

**Table 3:** Properties of traditional phenolic foam (PF) and date palm bio-oil substituted phenolic foam (DPF).

	Density (kg/m^3^)	Thermal Conductivity (W/m·K)	Compressive Strength (kPa)
PF	49 ± 1	0.035 ± 0.001	105 ± 7
10DPF	73 ± 1	0.040 ± 0.001	202 ± 5
15DPF	90 ± 2	0.038 ± 0.002	294 ± 2
20DPF	61 ± 1	0.036 ± 0.002	146 ± 10

**Table 4:** Cell structure properties of traditional phenolic foam (PF) and date palm bio-oil substituted foam (DPF).

	Density (kg/m^3^)	Minimum Cell Size (μm)	Maximum Cell Size (μm)	Mean Cell Size (μm)	Minimum Cell Thickness (μm)	Maximum Cell Thickness (μm)	Mean Cell Wall Thickness (μm)
PF	49 ± 1	25	188	85.9 ± 34	4.5	39.5	12.6 ± 7
10DPF	73 ± 1	9	210	57.09 ± 45	9.4	44.2	21.7 ± 7
15DPF	90 ± 2	9	188	54.4 ± 36	5	45.6	28.6 ± 11
20DPF	61 ± 1	18	150	64.5 ± 32	9.8	31.6	22.3 ± 6

**Table 5:** TGA and DTG data of traditional phenolic foam (PF) and date palm bio-oil substituted phenolic foams (DPFs).

	T-_5%_ (°C)	T_max_ (°C)	Residual Weight (%)			
			200 °C	400 °C	600 °C	800 °C
PF	124.7	564.2	91.6	81.9	52.2	29.1
10DPF	180.9	587.4	93.	84.6	56.5	32.4
15DPF	192.1	588.9	94.4	84.1	55.7	30.6
20DPF	147.3	567.	92.5	83.4	54.1	30.1

## Data Availability

The raw/processed data required to reproduce these findings cannot be shared at this time as the data also form part of an ongoing study.
